# New additional scoring formula on the Pathological Features in Stage I Lung Adenocarcinoma Patients: Impact on Survival

**DOI:** 10.7150/ijms.45002

**Published:** 2020-07-19

**Authors:** Shilei Zhao, Fengzhou Li, Xin Guo, Tao Guo, Ken-ichi Mizutani, Sohsuke Yamada, Chundong Gu, Hidetaka Uramoto

**Affiliations:** 1 Department of Thoracic Surgery, the First Affiliated Hospital of Dalian Medical University, Dalian, Liaoning, China.; 2 Department of Pathology and Laboratory Medicine, Kanazawa Medical University, Kanazawa, Ishikawa, Japan.; 3 Department of Thoracic Surgery, Kanazawa Medical University, Kanazawa, Ishikawa, Japan.

**Keywords:** Prognosis, Pathological classification, Lung adenocarcinoma, Heterogeneity

## Abstract

**Background:** Histological heterogeneity of lung adenocarcinoma may result in different prognosis among patients with the same TNM pathological stage. However, no objective evaluation system of lung adenocarcinoma based on pathological features has been widely accepted for assessing the prognosis.

**Methods:** We retrospectively analyzed 179 patients with stage I lung adenocarcinoma after complete surgical resection. The pathological classification was according to the IASLC/ATS/ERS adenocarcinoma classifications, and the detailed abundance ratio using HE staining of primary tumor specimens was recorded. A new additional scoring formula on the pathological features (ASP) was established. The association of the ASP score with the patients' survival was examined.

**Results:** The ASP scoring was significantly associated with smoking history (p=0.004), lymphatic vessel invasion (p<0.001), vascular invasion, differentiation (p<0.001) and Ki67 (p<0.001). The patients in the high-ASP-score group tended to have vascular invasion (odds ratio [OR]: 1.637, 95% confidence interval [CI]: 1.923-13.745, p=0.001) and high Ki67 expression (OR: 2.625, 95%CI: 1.328-5.190, p=0.006) by logistic regression analyses. The prognosis differed significantly in the Kaplan-Meier survival curves, and the 5-year survival rates in the low and high ASP score groups were 97.8% and 89.6%, respectively (p=0.018). Based on the univariate analysis, female (OR: 0.111, 95%CI: 0.014-0.906, p=0.040), long smoking history (OR: 7.250, 95%CI: 1.452-36.195, p=0.016), poor differentiation characteristics correlation (OR: 12.691, 95%CI: 1.557-103.453, p=0.018), and high ASP score (OR: 5.788, 95%CI: 1.138-29.423, p=0.034) were shown to be independently associated with an unfavorable prognosis.

**Conclusion:** The ASP score can effectively screen high-risk patients for complete surgical resection of stage I lung adenocarcinoma.

## Introduction

In recent years, adenocarcinoma has ranked as the most important pathological type of lung cancer. As such, early screening programs for high-risk populations have been developed, and its incidence is increasing gradually year by year around the world 1,2,3. Although the diagnosis and treatment of lung cancer continues to progress, resulting in an improved prognosis 4,5 lung adenocarcinoma, as a representative of tumor heterogeneity, has a poor prognosis due to its characteristics of early metastasis and recurrence 6,7. Even in stage I lung adenocarcinoma, the five-year survival rate is still 70%, and 30% high-risk patients tend to experience tumor progression 8,9,10. Therefore, how to differentiate the high-risk group from stage I lung adenocarcinoma patients after surgery has become an urgent clinical problem to be solved.

In clinical practice, evidence supporting the further treatment of patients after complete surgical resection of stage I lung adenocarcinoma is lacking 11. However, we frequently encounter various pathological subtypes from the same lung adenocarcinoma lesion, and its proportion in tumor tissue is also discrepant, due to the tumor's inherent histological heterogeneity. With the introduction and subsequent popularization of the International Association for the Study of Lung Cancer (IASLC), the American Thoracic Society (ATS), and the European Respiratory Society (ERS) lung adenocarcinoma classification systems since 2011 12, most literature has shown that the prognosis of stage I lung adenocarcinoma patients differs based on the predominant pathological subtype 13,14,15. However, despite these findings, detailed information on pathological mixed subtypes and the proportion of different morphological structures in the focus of adenocarcinoma has not been attracted sufficient attention. This may be the breakthrough point for identifying high-risk patients from these who the same pathological TNM staging, especially in early lung cancer.

At present, no objective system for evaluating lung adenocarcinoma has been widely accepted, in stark contrast to the situation with other tumors, such as breast, prostate, and kidney cancers 16,17,18. Therefore, the present study explored a novel pathological risk scoring system of stage I lung adenocarcinoma based on pathological subtypes and the abundance ratio using hematoxylin-eosin staining. We also examined the frequency and clinical significance of additional scoring formula on the pathological features (ASP) in a retrospective series of 179 patients resected for adenocarcinoma of the lung and analyzed its impact on patients' prognosis. Our results indicate that ASP could facilitate the differentiation of high-risk patients from lower-risk ones and leading to appropriate early postoperative intervention.

## Methods

### Patients and follow-up

A total of 244 consecutive patients who underwent radical surgery of the primary tumor and systematic lymph node dissection or sampling at the Department of Thoracic Surgery of Kanazawa Medical University from January 2005 to December 2015 were included in this study. Of these, 179 patients (median age: 68.7 years old; range: 37-83 years old) were confirmed to have stage I lung adenocarcinoma by postoperative pathology (referring to the eighth edition tumor node metastasis [TNM] classification for NSCLC), and all had complete clinical data available with none receiving chemotherapy or radiotherapy prior to the operation. The hospital institutional review board of Kanazawa Medical University approved the protocol.

All enrolled patients were suggested to follow up according to our previous study protocol. The postoperative follow-up time points were every three months within the first year and every six months thereafter. During the follow-up, a physical examination, chest radiography, analysis of blood chemistry and carcinoembryonic antigen assay were performed. If any symptoms or signs of recurrence appeared in these examinations, further evaluations to detect the recurrent site were carried out. Follow-up time was terminated in May 2017 (median follow-up: 46 months).

### Histopathological evaluations

Formalin-fixed, paraffin-embedded, 3-μm-thick sections were obtained from 179 samples stained with hematoxylin and eosin for the histopathologic diagnosis. Of these, 144 cases had their pathological subtype and abundance ratio (over 5%) from the maximal tumor diameter cross section recorded in the pathological report by two pathologists with a single blind method; the remaining cases lacking detailed pathological information were read again by the senior professor (Dr. Yamada) in order to obtain complete pathological data.

Lung adenocarcinomas were classified according to the IASLC/ATS/ERS Classification into the following subtypes: adenocarcinoma *in situ* (AIS), minimally invasive adenocarcinoma (MIA), lepidic (Lep), acinar (Aci), papillary (Pap), micropapillary (MP), solid (Sol) and mucinous (Mc) adenocarcinoma. Other relevant information was obtained from the surgical pathology report or our follow-up database.

### Pathological risk scoring system

**Figure 1:**
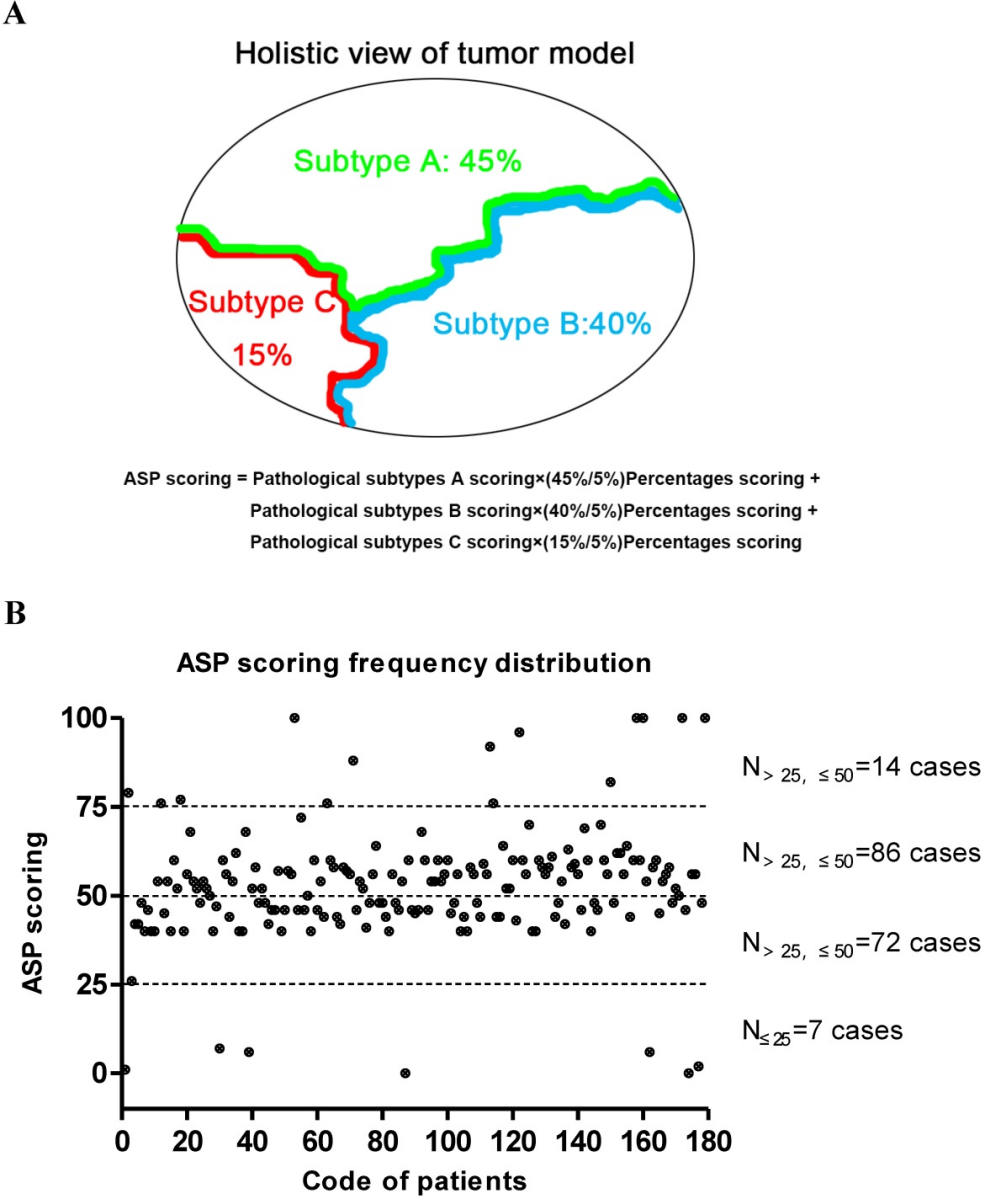
The Chart of ASP scoring pattern and its distribution in 179 patients with stage I lung adenocarcinoma. (A) A diagram of the formula on ASP scoring system. (B) The cumulative frequency curve chart of the ASP scoring distribution based on the pathological subtype and percentage content in 179 patients with stage I lung adenocarcinoma. The median ASP score was 52.7±16.3 points.

**Figure 2:**
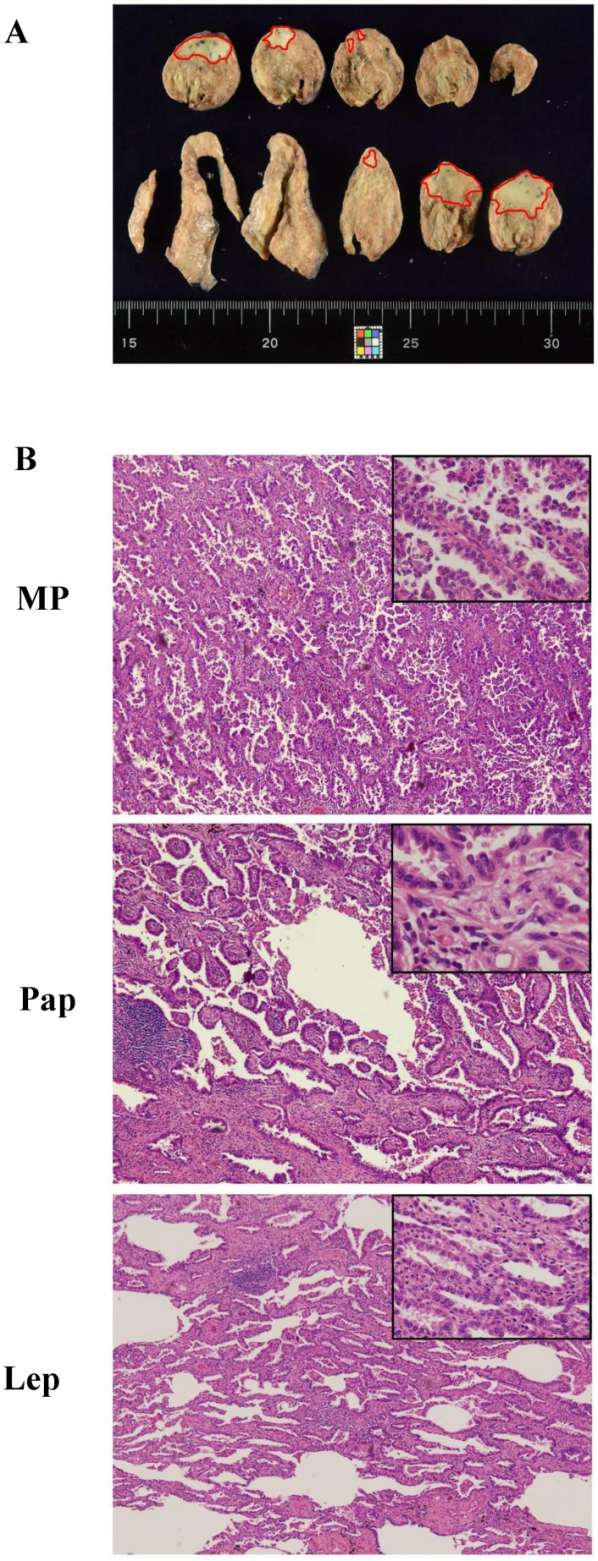
A typical case of mixed adenocarcinoma. (A) Focal area in the naked eye view, marked with a red dot. (B) The whole tumor contains lepidic (Lep; 40%), papillary (Pap; 50%), and micropapillary (MP; 10%) growth patterns (magnification 40× and 100x). The ASP score of this patient was 2 points × (40%/5%) + 3 points × (50%/5%) + 5 points × (10%/5%).

The final overall appraised ASP score of each specimen was determined as the pathological subtype points multiplied by the percentage content points. Adenocarcinoma *in situ* (AIS) was classified as a precancerous lesion and its 5-year overall survival rate was nearly 100%. In its strictest sense, it was not belong to cancer. So we defined the pathological subtype points of AIS was 0 point. Minimally invasive adenocarcinoma (MIA) belong to adenocarcinoma mainly has a lepidic growth pattern, but its invasive component was less than 5mm. Furthermore, it had better prognosis as well as AIS. Therefore, the patients with MIA was diagnosed clinically, the pathological subtype points of MIA was defined as 1 point regardless of the invasive growth pattern. Other invasive adenocarcinomas include Lep, Aci, Pap, MP, Sol and Mc were given the different pathological subtype points according to their prognosis 19,20. The worse the prognosis, the higher score. The specific scoring criteria and pattern are shown in Table 1 and Figure 1A. The distribution of the total score from 179 patients with stage I lung adenocarcinoma is shown in Figure 1B, and representative case contemporary contains three different growth patterns is shown in Figure 2A and 2B.

### Statistical analysis

The association between the ASP score distribution interval and clinicopathologic characteristics was assessed by Pearson's chi-squared test. For the correlation analysis of the different ASP score and multiple variables, logistic regression analyses was constructed by the backward selection of multiple variables. Survival curves were calculated using the Kaplan-Meier method. The log-rank test was used to analyze the overall survival time by ASP scores in stage I lung adenocarcinoma. A univariate analysis was performed using the Cox regression model. Data were analyzed using the SPSS 22 software program (SPSS Inc, Chicago, IL, USA). Values of P<0.05 were considered statistically significant.

## Results

### Clinicopathologic characteristics correlation between the ASP score distribution and clinicopathologic variables

A total of 87 (48.6%) of the 179 patients with complete surgical resection of stage I lung adenocarcinoma were male, and 92 (51.4%) were female. Among the 179 patients, 64 (35.8%) were former or current smokers, and the smoking index exceeded 400. In 66 patients (36.9%), the expression of the tumor proliferation bio-marker Ki-67 exceeded 10%. The numbers of cases in the right upper, right middle, right lower, left upper and left lower lobes were 60 (33.5%), 20 (11.2%), 33 (18.4%), 40 (22.4%) and 26 (14.5%), respectively. The surgical procedure employed was open thoracotomy in 9 patients (5.0%), complete video-assisted thoracic surgery [c-VAST] thoracotomy in 2 patients (1.1%) and hybrid resection in 168 patients (93.9%). Tumors were classified as well, moderately and poorly differentiated in 107 (59.8%), 60 (33.5%) and 12 (6.7%) cases, respectively. Vascular and lymphatic invasion was observed in 48 (26.8%) and 54 (30.2%) cases, respectively.

### Correlation between the ASP score distribution and clinicopathologic variables

Patients were divided based on their ASP score into a low-score group (≤50 points; n=79 [44.1%]) and high-score group (>50 points; n=100 [55.9%]) (Table 1). The ASP score distribution was significantly associated with the smoking history (p=0.004), lymphatic vessel invasion (p<0.001), vascular invasion, differentiation (p<0.001) and Ki67 (p<0.001), as seen in Table 2, but not with the gender, age, location or maximum tumor diameter. Furthermore, patients with higher ASP scores tended to have poor differentiation, vascular or lymphatic invasion and abnormally elevated Ki67 expression, which often indicate a poor prognosis.

Subsequently, we found that ASP score categories were correlated with vascular invasion and high Ki67 expression using by logistic regression analysis. These values are shown in Table 3. In addition, we found that patients in the high-score group for ASP tended to have vascular invasion (odds ratio [OR]: 1.637, 95% confidence interval [CI]: 1.923-13.745, p=0.001) and high Ki67 expression (OR: 2.625, 95%CI: 1.328-5.190, p=0.006) than those in the low-score group.

### Survival analyses

**Figure 3:**
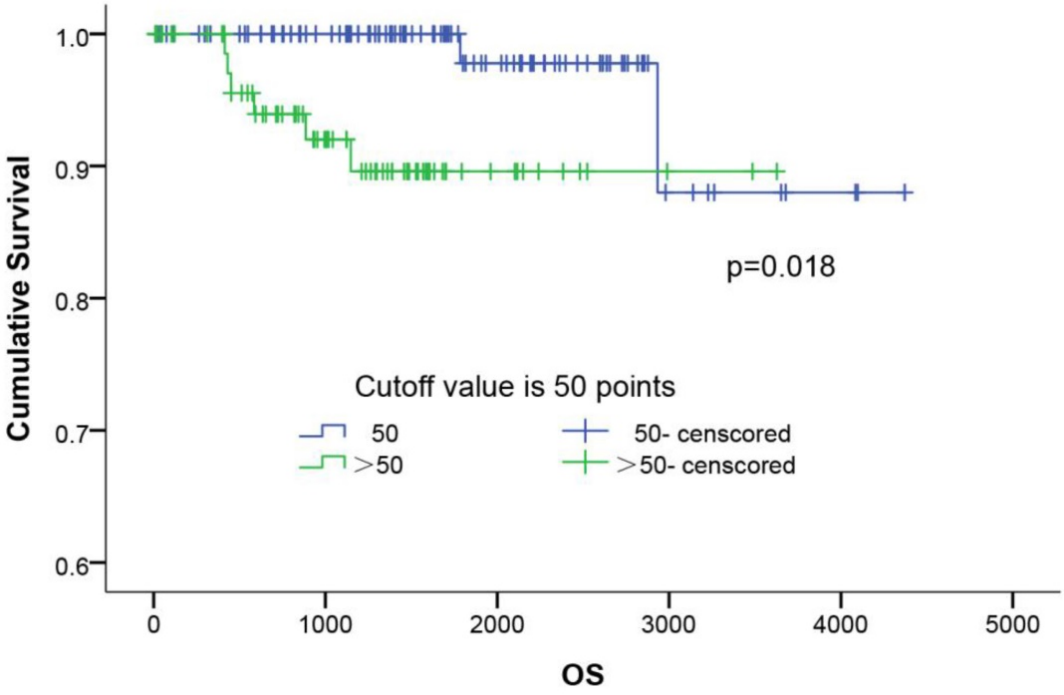
The survival curves of ASP scoring system. The survival curves of ASP scoring in 179 patients with completely resected stage I lung adenocarcinoma using the Kaplan-Meier method. The 5-year survival rates of the low (≤50), and high ASP score (>50) groups was 97.8% and 89.6%, respectively (p=0.018).

The influence of the ASP score distribution on the patients' overall survival (OS) was evaluated. As shown in Figure 3, the Kaplan-Meier survival curves of all 179 patients showed that the patients with a higher ASP score (P=0.018) had a shorter OS than those with a lower score. The 5-year survival rates in the low- (≤50) and higher-ASP-score (>50) groups were 97.8% and 89.6%, respectively. Further, a multivariate Cox proportional hazards model analysis (Table 4) of the stage I lung adenocarcinoma patients indicated that female (OR: 0.111, 95%CI: 0.014-0.906, p=0.040), long smoking history (OR: 7.250, 95%CI: 1.452-36.195, p=0.016), poor differentiation (OR: 12.691, 95%CI: 1.557-103.453, p=0.018), and high ASP score (OR: 5.788, 95%CI: 1.138-29.423, p=0.034) were significant risk factors for predicting poor prognosis.

## Discussion

In the present study, lung adenocarcinoma was frequently encountered in the form of a malignant mixed tumor, including two or more pathological subtypes, and these pathological subtypes showed obvious differences in tumor progression. This phenomenon, known as intra-tumor heterogeneity, may result in different prognoses among similar lung adenocarcinoma patients 21,22,23. Indeed, Sica et al.^24^ and Boland et al. 25 reported that the predominant combinations of pathological subtypes are associated with different prognoses. Although radiotherapy, chemotherapy and molecular-targeted therapy have improved the prognosis of patients with lung adenocarcinoma, 30% of patients with early lung adenocarcinoma still develop tumor metastasis or recurrence after undergoing radical surgery 26. It has been suggested that the response to adjuvant therapy is unevenly distributed among these patients. Therefore, it is particularly important to distinguish patients at a high risk of recurrence and metastasis from seemingly identical patients who have a lower risk of such outcomes.

Many studies have shown that the assessed differing expression of tumor genes using immunohistochemical staining can effectively predict the prognosis in lung cancer 27, 28, but these bio-markers are only expressed in a certain subgroup of tumor cells and are measured according to pathologists' subjective experience. Furthermore, pathologists from different laboratories can draw opposing conclusions in the same case due to variations in sample processing and experimental techniques. Multilocus detection using second-generation sequencing technology has gradually developed 29, 30, but the costliness of such testing and low device penetration limit its application. In contrast, the approach proposed in the present study has advantages of simple controllability and good practicability. While this process may be time-consuming and laborious, a new additional scoring formula on the pathological features (ASP) reveals crucial information on patients' lesions more comprehensively and accurately, thereby facilitating the differentiation of high-risk patients from lower-risk ones and leading to appropriate early postoperative intervention, and which can be carried out smoothly in most primary-care hospitals. Furthermore, this scoring system also has prognostic implications, as it is capable of stratifying patients into different risk-rank groups for the OS of stage I lung adenocarcinoma and predicting the outcomes of individual patients after an operation. It therefore has potential utility in guiding disease management, similar to what is already being practicing in other organ systems.

Among the total 179 patients with stage I lung adenocarcinoma after complete surgical resection, patients with higher ASP scores tended to have poorly differentiation (p<0.001), vascular or lymphatic invasion (p<0.001) and abnormally elevated Ki67 expression (p<0.001), factors that are closely associated with a poor prognosis. This suggests that the ASP score may be more characteristic in evaluating patients' tumor status. Subsequently, the Kaplan-Meier survival curves also showed that the patients with higher ASP scores (P=0.018) had a shorter OS than those with lower scores. However, cytology or needle aspiration biopsy may not be appropriate for the ASP system that dependent on an adequately sampled resected tumor. While the nuclear morphology and chromatin mitotype usually in estimating of tumor malignant degree were not considered in this scoring system, but ASP was related to vascular invasion and Ki67 expression may indirectly reflect the degree of tumor malignancy to some extent. In the univariate analysis we found the significance of ASP score, but not appeared in the subsequent multivariate analysis (no listed in text). With the increased number of cases and the other clinical features, we expect the ASP system will be applicable not only to phase I lung cancer but also other stages of lung adenocarcinoma in the future. At present, technology supporting an artificial intelligence (AI) diagnosis is developing rapidly; 31, 32 such an approach may reduce the error-bias of supervisor judgment concerning the pathologic diagnosis and save labor costs. However, it may be some time before a mature algorithm and mathematics model can be established. As a short-term solution, the ASP score system for assessing the prognosis of patients can be implemented in basic-level hospitals, although it is admittedly time-consuming and laborious to use. Our method of evaluating whole pathological sections may be used to generate references for an AI prototype algorithm. However, the disease-free survival is more significant than the overall survival we used in predicting the early recurrence and metastasis of lung adenocarcinomas, but we only obtained the cause of death and time of the patients via institutional investigation that was limitation of our study. Therefore, the ASP scoring system needs to be further improved in the follow-up studies, including the acquisition of patients' disease-free survival.

## Conclusion

In conclusion, the ASP score can effectively screen high-risk patients for complete surgical resection of stage I lung adenocarcinoma and help inform the early postsurgical management of these patients.

## Figures and Tables

**Table 1:** Points assigned for different pathological subtypes and percentages for lung adenocarcinoma

Score	0 point	1 point	2 point	3 point	4 point	5 point
Pathological subtypes	AIS	MIA	Lep	Aci&Pap	Mc	MP&Sol
Percentages (%)	1 point per 5%					

Abbreviations: adenocarcinoma *in situ* (AIS), minimally invasive adenocarcinoma (MIA), lepidic adenocarcinoma (Lep), acinar adenocarcinoma (Aci), papillary adenocarcinoma (Pap), micropapillary adenocarcinoma (MP), solid adenocarcinoma (Sol) and mucinous adenocarcinoma (Mc).

**Table 2:** Relationship between the ASP scoring frequency distribution and clinicopathologic characteristics

Factor	Low scores			High scores			p-value
	≤25	>25 to 50	N1=79	>50 to 75	>75	N2=100	
**Gender**							0.054
male	1	31	32 (49.4%)	44	11	55 (50.6%)	
female	6	41	47 (64.1%)	42	3	45 (35.9%)	
**Age, years**							0.341
≤68.7	3	39	42 (47.7%)	43	3	46 (52.3%)	
>68.7	4	33	37 (44.1%)	43	11	54 (55.9%)	
**Smoking history**							0.004
<400	6	54	60 (52.2%)	53	2	55 (47.8%)	
≥400	1	18	19 (29.7%)	33	12	45 (70.3%)	
**Location (lobe)**							0.674
right-up	2	28	30 (63.3%)	26	4	30 (36.7%)	
right-mid	1	6	7 (50.0%)	10	3	13 (50.0%)	
right-down	2	11	13 (45.5%)	17	3	20 (54.5%)	
left-up	0	19	19 (67.5%)	18	3	21 (32.5%)	
left-down	2	8	10 (46.2%)	15	1	16 (53.8%)	
**T max**							0.686
≤1cm	4	1	5 (71.4%)	2	0	2 (28.6%)	
>1 cm to 2 cm	30	15	45 (58.4%)	20	12	32 (41.6%)	
>2 cm to 3 cm	14	17	31 (51.7%)	22	7	29 (48.3%)	
>3 cm	12	9	21 (60.0%)	4	10	14 (40.0%)	
**Lymphatic vessel invasion**							<0.001
Absent	7	61	68 (54.4%)	47	10	57 (45.6%)	
Present	0	11	11 (20.4%)	39	4	43 (79.6%)	
**Vascular invasion**							<0.001
Absent	7	67	74 (56.5%)	50	7	57 (43.5%)	
Present	0	5	5 (10.4%)	36	7	43 (89.6%)	
**Differentiation**							<0.001
High	7	69	76 (71.0%)	30	1	31 (29.0%)	
Moderate	0	3	3 (5.0%)	54	3	57 (95.0%)	
Poor	0	0	0 (0.0%)	2	10	12 (100.0%)	
**Ki67**							<0.001
≤10%	6	61	67 (59.3%)	43	3	46 (40.7%)	
≥10%	1	11	12 (18.2%)	43	11	54 (81.8%)	

**Table 3:** Results of logistic regression analyses on patients with high or low ASP scoring and multiple variables

Variable	OR	95% CI	p-value
**Smoking history**			
≥400	1		
<400	0.974	0.963-1.002	0.312
**Lymphatic vessel invasion**			
Present	1		
Absent	0.571	0.526-3.200	0.571
**Vascular invasion**			
Absent	1		
Present	1.637	1.923-13.745	0.001
Differentiation			
Moderate & poor	1		
High	0.885	0.638-1.229	0.466
Ki67			
<10%	1		
≥10%	2.625	1.328-5.190	0.006

**Table 4:** Univariate analysis of overall survival.

Variable	OR	95% CI	p-value
Age, years			
≤68.7	1		
>68.7	12.489	0.349-43.794	0.108
Gender			
Male	1		
Femal	0.111	0.014-0.906	0.040
Smoking history			
<400	1		
≥400	7.250	1.452-36.195	0.016
Operation method			
c-VATS	1		
Hybrid&Open	3.465	0.635-18.891	0.151
ASP scoring			
Low	1		
High	5.788	1.138-29.423	0.034
T max			
≤3cm	1		
>3 cm	3.352	0.835-13.466	0.088
Lymphatic vessel invasion			
Absent	1		
Present	0.380	0.046-3.116	0.367
Vascular invasion			
Absent	1		
Present	1.283	0.254-6.493	0.763
Differentiation			
High	1		
Moderate&Poor	12.691	1.557-103.453	0.018
Ki67			
<10%	1		
≥10%	2.389	0.583-9.790	0.226

## Abbreviations

IASLC: the International Association for the Study of Lung Cancer
ATS: the American Thoracic Society
ERS: the European Respiratory Society
ASP: additional scoring formula on the pathological features
AIS: adenocarcinoma *in situ*
MIA: minimally invasive adenocarcinoma
Lep: lepidic adenocarcinoma
Aci: acinar adenocarcinoma
Pap: papillary adenocarcinoma
MP: micropapillary adenocarcinoma
Sol: solid adenocarcinoma
Mc: invasive mucinous adenocarcinoma
AI: artificial intelligence
